# A ‘health system’ perspective on scaling up hospital cataract services

**Published:** 2015

**Authors:** Tazeem Bhatia

**Affiliations:** Specialty Registrar in Public Health: Disability and Eye Health Group, London School of Hygiene and Tropical Medicine, London, UK. Email: **tazeem.bhatia@lshtm.ac.uk**

**Figure:**
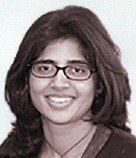
Tazeem Bhatia

Even though cataracts are treatable, they are still a leading cause of blindness in low- and middle-income countries.^1^ This is due to supply and demand factors including quality of services, cost, availability and lack of awareness in the community.

## What does it take to perform a single cataract operation?

If we want to set up a cataract surgical programme, we might first think about needing a trained surgeon, the right equipment and trained support staff. But we should also ask questions like:

- Where would the operations take place? The facility would need a reliable source of electricity and water.
- How will the programme be funded, and who will pay for the staff, the equipment and the consumables?
- How could we ensure that staff have the right qualifications and skills?
- How will equipment and consumables be procured, and where will they be stored and maintained?
- How will we ensure that people come to us for surgery?

Although surgery to treat cataracts and the associated visual impairment or blindness isa straightforward intervention, establishing an effective programme requires a wider infrastructure or system to support it, which is known as the eye health system. Essentially, it requires much more than a surgeon to perform the cataract operation and it is the strengthening of this wider infrastructure that is key to increasing the availability and uptake of cataract services.

**Figure 1.**
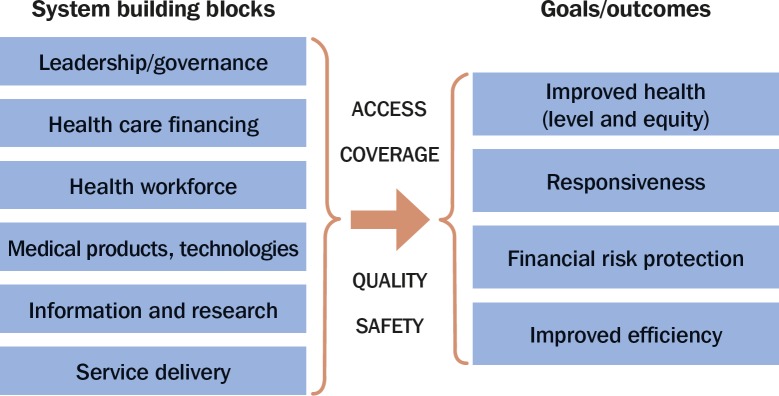
The WHO Health Systems Framework^1^

## How does this relate to cataract services?

According to the World Health Organization (WHO) a health system comprises six building blocks that are not separate, but relate to each other (see Figure 1). These blocks can be used as a framework for understanding an eye health system and defining the priority areas that need to be further developed.

If we analyse a hospital cataract surgical programme using the six blocks, how would it look? The following are examples of the problems and solutions that could be identified by using the six building blocks framework.

1. **Governance** is about how decisions are made. Not just a bout who makes them, but also about how much influence the community, patients and staff have in making the cataract service relevant and a positive experience for them. An effective programme depends on the active participation and partnership of all these people.
2. **Health financing.** It is important to know how much the cataract surgical programme costs and how much revenue it generates. What is the shortfall? What are the alternatives sources of funding? Regular monthly and quarterly reviews allows us to predict when there might be gaps in funding so we can advocate for extra funds or plan services differently to avoid interruptions.
3. **Health workforce.** Are there enough trained personnel? If not, is this because there are not enough personnel being trained nationally or is there an uneven distribution of personnel across the country or region? Solutions could be on-the-job training of existing staff or advocating nationally for the training and accreditation of staff.
4. **Medical products** make up a large percentage of the cost of services for patients. Cost is a main barrier for many people, so finding ways to reduce costs is important. Drugs can be procured nationally through a national procurement process or bought in bulk by a group of hospitals working together; all of which drives costs down.
5. **Information and research.** What eye health information is collected and how is it used? The only way to understand whether a service is doing what it was set up to do is by collecting and analysing the activity and outcome data. This means not just how many cataracts were identified and operated on, but whether the patients could see better after the operation. Having good quality outcomes is important as it helps to justify further funding; it also gives the service a good reputation in the community.
6. **Service delivery.** What policies and processes are in place to reduce the number of adverse outcomes, for example post-operative infection? Improving service delivery could mean monitoring hand washing or the sterilisation process for theatre and the effect that better infection control has on the post-operative infection rate.

These are just examples of how the building blocks can be applied and used to understand the strengths and weaknesses in an existing cataract surgery programme. The blocks could also be used as a framework for ensuring that all aspects of planning a new cataract service have been thought through.

Breaking down the elements of an eye health system into the six areas is a simple way of categorising the issues and helps to generate a priority list of actions to strengthen the system. It is through strengthening the eye health system that we can build resilient and sustainable programmes. However, it is important to have a whole-system perspective and not to address individual areas separately. Remember that this is a complex, dynamic system in which everything interrelates.^2^
